# Mucus-Inspired Supramolecular Adhesives: Exploring the Synergy between Dynamic Networks and Functional Liquids

**DOI:** 10.1021/acsnano.5c02399

**Published:** 2025-04-14

**Authors:** Changshun Hou, Wenqing He, Xi Yao

**Affiliations:** † Department of Biomedical Sciences, City University of Hong Kong, Hong Kong SAR 999077, P. R. China; ‡ Shenzhen Research Institute, City University of Hong Kong, Shenzhen 518000, P. R. China

**Keywords:** mucus, bioinspired material, supramolecular polymer, gel, hydrogen bond, micro/nanochannel, wearable sensor

## Abstract

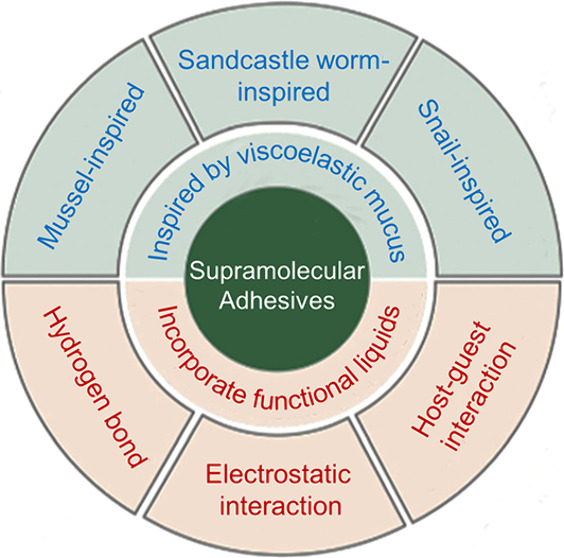

The exceptional physicochemical and mechanical properties of mucus have inspired the development of dynamic mucus-based materials for a wide range of applications. Mucus’s combination of noncovalent interactions and rich liquid phases confer a range of properties. This perspective explores the synergy between dynamic networks and functional liquids in mucus-inspired supramolecular adhesives. It delves into the biological principles underlying mucus’s dynamic regulation and adhesive properties, the fundamentals of supramolecular adhesive design, and the transformative potential of these materials in biomedical applications. Finally, this perspective proposes potential directions for the molecular engineering of mucus-inspired supramolecular materials, emphasizing the need for interdisciplinary approaches to harness their full potential for biomedical and sustainable applications.

Adhesives that can bind to diverse synthetic and biological surfaces in various environments have great technical implications in areas ranging from aquatic vehicles, and soft robotics to wound dressings and biomedical devices.^1−3^ For example, adhesives that can provide robust underwater adhesion can be used as sealants for emergency water leak repair, and biocompatible adhesives that can bond strongly to biological tissues have great potential for wound dressing and organ repairing.^4−6^ With such strong demands, intensive efforts have been devoted to the development of adhesive materials in the past decade, with a particular focus on hydrogels or hydrogel-derived adhesives.^7−9^ However, adhesive materials need to maintain functions under complex and changeable environments and their performance needs to be durable or adjustable to meet specific application demands, imposing high-level requirements on the material innovation and development.

In the quest to develop innovative materials, nature often serves as a profound source of inspiration.^10,11^ In nature, there are abundant adhesive interfaces with adaptive performance regulated by specific structural or molecular mechanisms,^12−14^ such as the suction cup of octopuses,^15^ the hierarchical structures of gecko toes,^16,17^ the patterns of tree frog toes,^18^ and the mussel adhesives.^19^ Among the myriads of biological adhesive systems, mucus stands out as a remarkable example due to its dynamic regulation, multifunctionality, and impressive adhesive behavior.^20,21^ Several organisms utilize mucus for adhesive purposes in their natural habitats. For instance, gastropods such as snails and slugs rely on mucus to anchor themselves to surfaces, even under challenging environmental conditions.^22,23^ Similarly, marine organisms like sandcastle worms employ protein-based polyelectrolytes for complex coacervation, producing liquid–liquid phase separation for reversible underwater adhesion.^8,24^ Mussels use mucus-like adhesive secretions to attach to underwater surfaces,^25^ demonstrating the natural material’s versatility and robustness. All of these natural adhesive phenomena provide valuable insights into the design of synthetic materials.^26^

In the human body, mucus serves as a dynamic and multifunctional lining layer to cover epithelial layers of human organs such as the respiratory tract, gastrointestinal tract, reproductive system, and ocular surface.^27^ It is continuously produced, secreted, transported, recycled, and discarded and therefore performs a variety of vital functions, including lubrication, protection, filtration, and adhesion. All of them are closely related to the physiological condition of the human body. On the one hand, these features not only maintain homeostasis but also play a crucial role in disease pathology and drug-delivery challenges.^28^ On the other hand, its ability to adapt to different environmental conditions and maintain functionalities makes mucus an exceptional model for bioinspired materials, particularly gels and adhesives.^29^ Understanding the biological composition and structure of mucus is essential to unlocking its potential as a template for the development of advanced synthetic materials in drug delivery, tissue engineering, medical implants, and wearable devices.

At the core of mucus’s multifunctionality and adhesive properties are its dynamic polymeric networks and functional liquid components. Together, these elements create a synergistic system characterized by adaptability, selective permeability, and responsiveness to environmental stimuli. These attributes make mucus an ideal model for designing supramolecular adhesives, which rely on reversible, noncovalent interactions to achieve tunable and versatile bonding (Figure 1). By mimicking the natural synergy between dynamic networks and functional liquids, researchers may uncover transformative solutions for challenges in biomedicine and sustainability.

**Figure 1:**
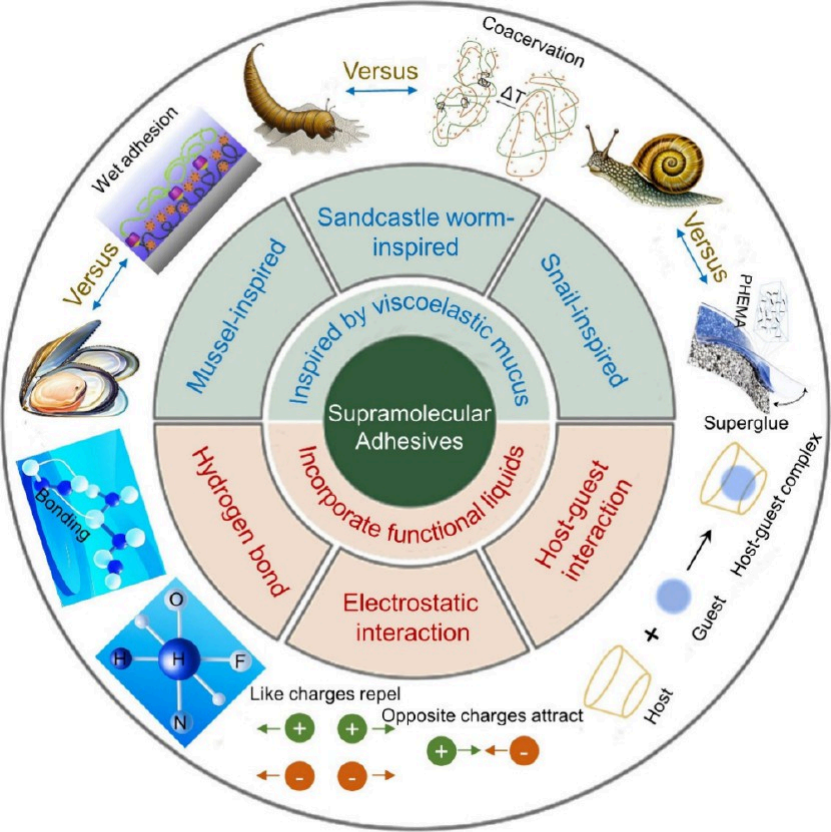
Inspired by viscoelastic mucus in different creatures, supramolecular adhesives can utilize various noncovalent interactions to develop the required properties. One supramolecular interaction or multiple supramolecular interactions can be introduced to one adhesive system. Optical images of a mussel, sandcastle worm, and snail are produced with a completely free Cici-AI photographer. The mussel-inspired structure (a dopamine-modified supramolecular adhesive) is adapted with permission under a Creative Commons License from ref (30). Copyright 2017 Springer Nature. The snail-inspired structure (an intrinsically reversible polymeric superglue) is adapted with permission from ref (23). Copyright 2019 National Academy of Sciences.

This perspective explores the synergy between dynamic networks and functional liquids in mucus-inspired supramolecular adhesives. It gives a critical review delving into the biological principles underlying mucus’s dynamic regulation and adhesive properties, the fundamentals of supramolecular adhesive design, and the potential applications of these adhesive materials. By examining the intersection of biology and material science, this perspective highlights how mucus-inspired designs can address pressing needs for innovative adhesive technologies in an array of fields. The discussion underscores the transformative potential of mucus-inspired adhesives in biomedical and sustainable applications, focusing on specific innovations, their underlying mechanisms, and the challenges involved in their development.

## Understanding Mucus: A Biological Perspective

### Function, Composition, and Structural Dynamics of Mucus

**Figure 2:**
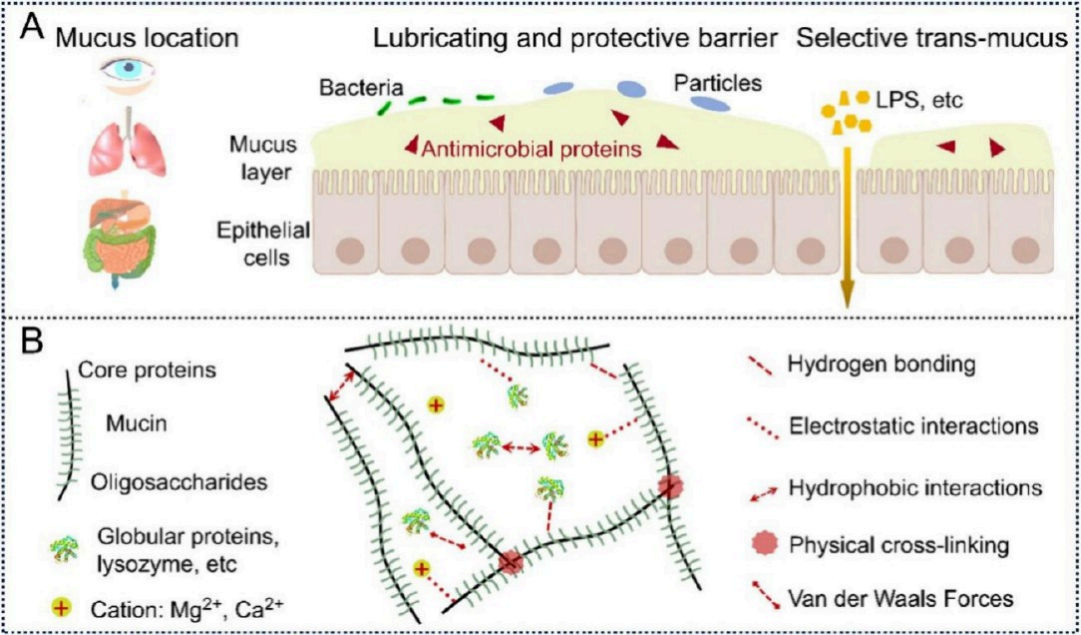
(A) Mucus is usually located in the eyes, lungs, and digestive system. It can work as a lubricant and protective barrier with both passive physical isolation and active antibacterial mechanisms. In addition, it offers the selective trans-mucus delivery of needed lipopolysaccharide (LPS) and ions. (B) The mucus is constructed from highly dynamic three-dimensional networks comprising water, mucins, globular proteins, and various ions, which are assembled together through abundant supramolecular interactions such as hydrogen bonding, electrostatic interactions, hydrophobic interactions, physical cross-linking, and van der Waals forces.

Mucus is a fascinating biological material that serves as a crucial interface between living organisms and their environment.^31^ It can be found in a diverse range of organisms, from humans to mollusks (Figure 2). For example, in human eyes, the mucus layer helps keep the conjunctiva lubricated, carries debris away, and comforts the eye between blinks. In the human lung, the mucus layer acts as a surfactant to lower and balance the surface tension of alveoli at diverse sizes, enabling smooth and robust gas exchange of the lung.^32^ It also serves as a barrier to protect the underlying epithelial tissue from the invasion of pathogens and foreign substances.

The thickness of mucus layers varies from organ to organ. In the respiratory tract, the thickness of a mucus layer is around tens of microns in the trachea and in the bronchi. Such small thickness can facilitate the clearance and expulsion of mucus from the lung and airway track via the mucociliary transport mechanism.^33^ The mucus layer becomes much thicker in the stomach and colon, and the thickness of gastrointestinal mucus can reach hundreds of microns to allow both the firm adhesion of mucosa and the protection against pathogen invasion.^34^ The destruction or overproduction of mucus is observed in a range of diseases, from cystic fibrosis to peptic ulcer and ulcerative colitis.^35^

Under almost all conditions, mucus is predominantly composed of water (>90%), mucins (0.2–5%), lipids, globular proteins, DNA, cellular debris, and various ions.^36^ These components form a slimy yet slippery layer that adheres to epithelial surfaces, creating a selective barrier. Mucins, the critical nonaqueous components, are high-molecular-weight glycoproteins that provide the structural framework of mucus through extensive glycosylation and polymerization.^37^ Mucins’ glycosylated domains interact with water molecules to form a hydrated network, while their hydrophobic and electrostatic interactions promote the formation of a dynamic mesh-like structure.^38^

The molecular structure of mucins enables mucus to form a three-dimensional network that is both elastic and fluid, or viscoelastic. This dynamic network structure is integral to mucus’s ability to respond to mechanical stress and environmental changes. At higher mucin concentrations, regions of dense polymer networks coexist with more fluid-like phases, creating a heterogeneous microenvironment. This phase separation enables mucus to balance its dual role as both a barrier and a selective filter, allowing for the controlled diffusion of nutrients, signaling molecules, and antimicrobial agents, while trapping harmful pathogens and particles.^39^

### Role of Dynamic Networks in Mucus Functionality

On the one hand, the mucin network’s pore size, which ranges from 20 nm to over 1 μm depending on the organ and physiological state, regulates the diffusion of molecules and micro/nanoparticles through mucus. For example, studies have shown that smaller nanoparticles (∼100 nm), such as viruses,^40^ can diffuse more freely through the mucus mesh, while larger or charged particles encounter greater resistance due to steric hindrance and electrostatic interactions.^41^ The rich and diverse polar motifs on the glycoproteins further enhance the retention of specific ions, molecules, and colloids. Such selective permeability is critical for the barrier’s protective function as well as poses challenges for drug-delivery systems in targeting mucosal tissues.^42^

Dynamic networks in mucus contribute to its adaptability, a feature that is particularly important in biological systems. The network’s ability to reorganize and recover from deformation is driven by noncovalent interactions such as hydrogen bonding, electrostatic forces, and hydrophobic interactions. These reversible interactions allow mucus to exhibit high adaptability, such as self-healing properties, making it an excellent candidate for bioinspired applications. For instance, the dynamic nature of mucus enables it to maintain its integrity even under mechanical stress,^43^ a property that is invaluable in designing adhesives for biomedical use.

### Role of Liquid Components in Mucus Functionality

On the other hand, the liquid component of mucus, primarily water, plays an equally important role. The liquid phase facilitates the mobility of the mucin network and supports the transport of ions and molecules. The hydration level within mucus directly affects its mechanical behavior, with higher water content resulting in a more fluid-like consistency. Conversely, reduced hydration leads to increased viscosity, which can be beneficial for effective barrier function.

Beyond its mechanical influence, the liquid phase plays a crucial role in the diffusion and transport of biomolecules, including nutrients, signaling molecules, and antimicrobial agents. The presence of dissolved ions and small solutes affects mucus’ ability to regulate pH and ionic strength, which is essential for maintaining homeostasis and supporting enzymatic activity.^44^ The controlled diffusion of molecules within the hydrated network allows for the selective passage of beneficial compounds while restricting harmful substances, enhancing mucus’s protective function.

Moreover, the interplay between the network and the liquid phase is crucial for mucus’s adhesive behavior.^21^ The liquid phase in mucus contains various ions and small molecules that interact with the mucin network to create an optimal environment for adhesion. These interactions are often mediated by ionic bonds and hydrophilic interactions, which allow mucus to adhere to a wide range of surfaces. For example, the presence of calcium ions in mucus has been shown to enhance its adhesive properties by cross-linking mucin chains.^45^ The dynamic bonds formed between mucins and the surrounding liquid enable mucus to have reversible adhesion. The interplay between the liquid and the network is a key feature that researchers can learn in the development of advanced synthetic adhesives.

The natural properties, composition, and functional characteristics of mucus (e.g., wet adhesion, self-healing, environmental responsiveness, antibacterial activity, and biocompatibility) offer great inspiration for the development of supramolecular adhesives. Specifically, the lubrication, barrier functions, and selective permeability of mucus serve as a foundation for engineering multifunctional synergistic adhesives. Furthermore, the dynamic supramolecular networks existed in mucus (e.g., hydrogen bonding, hydrophobic effects, and electrostatic interactions) provide a model reference for designing supramolecular adhesives with reversible adhesion, self-healing capabilities, and environmental responsiveness. The robust and reversible adhesion of mucus in wet environments could further provide inspirations to the development of smart adhesives with high adaptability and dynamic interfacial adhesion under various conditions. More detail on the design of supramolecular adhesives and the implementation of mucus-inspired design will be elaborated in the following sessions.

### Supramolecular Adhesives: Fundamentals and Design

Supramolecular chemistry is the study of molecular systems held together by noncovalent interactions.^46,47^ These interactions are typically much weaker than covalent bonds and, therefore, allow for dynamic assembly and disassembly of molecular structures. The principles of supramolecular chemistry emphasize molecular recognition, reversibility, and self-organization, making them ideal for applications where adaptability and responsiveness are critical.

Supramolecular adhesives have emerged as an exciting class of materials characterized by their dynamic, reversible bonding, and ability to adapt to complex surfaces and environmental conditions.^48^ Their development has been driven by a combination of advances in polymer chemistry, material science, and a deeper understanding of molecular interactions. This section explores the progress in supramolecular adhesives by focusing on four critical aspects: the chemical design of dynamic polymer networks, the role of reversible bonds in interfacial adhesion, the effect of polymer assembly on adhesive functionality, and the demonstration of tunability, reversibility, and multifunctionality in these systems.

**Figure 3:**
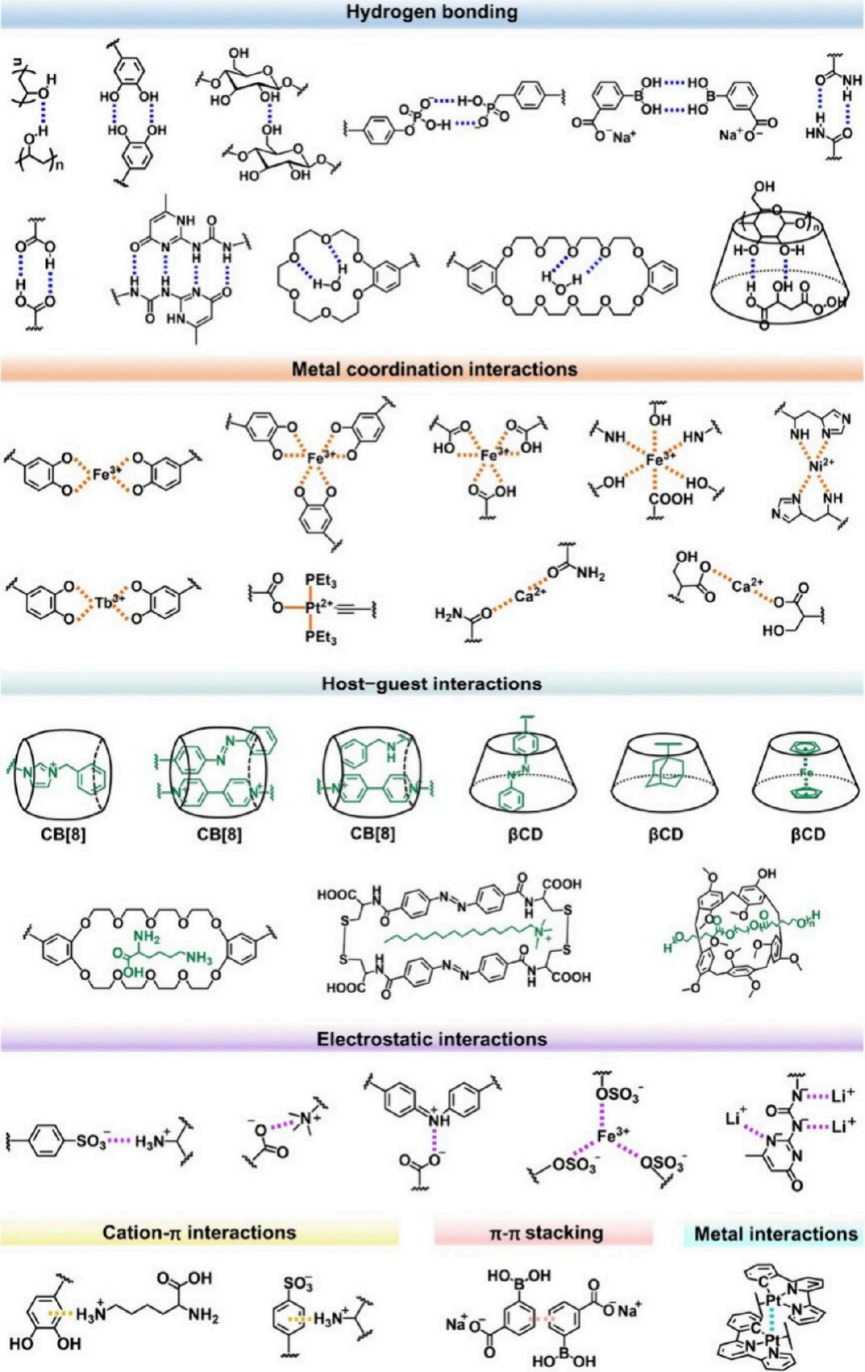
Different reversible bonds can be introduced in designing supramolecular adhesives. Reproduced with permission from ref (51). Copyright 2024 John Wiley and Sons.

The foundation of supramolecular adhesives lies in the design of polymer networks incorporating dynamic, reversible bonds. These bonds can include hydrogen bonds, metal–ligand coordination, π–π stacking, host–guest interactions, and ionic bonds (Figure 3), which enable the materials to form and re-form under specific conditions.^49^ The choice and arrangement of these bonding motifs directly influence the mechanical properties, adhesion strength, and environmental responsiveness of the adhesive.^50^

Recent efforts in chemical design have focused on creating hierarchical networks that combine multiple types of dynamic bonds.^52^ For example, integrating strong, reversible bonds (e.g., metal–ligand coordination) with weaker, rapidly reversible interactions (e.g., hydrogen bond) allows for the simultaneous optimization of strength and self-healing properties.^53^ Incorporating covalent adaptable networks, which use dynamic covalent bonds to add chemical stability while retaining reversibility, can further extend the capability of the adhesive to recyclability, reprocessability, and readhesion.^54^ These innovations have expanded the range of tunable properties, enabling adhesives to work in diverse environments, such as high humidity, extreme temperatures, or saline conditions.

Moreover, advances in polymer synthesis techniques, such as controlled radical polymerization and click chemistry, have allowed precise control over the molecular weight, architecture, and functional group distribution of the polymer network.^55^ This level of control has enabled researchers to design adhesives with predictable and programmable behaviors, further enhancing their applicability.

### Role of Reversible Bonds in Interfacial Adhesion

Interfacial adhesion is a critical property for any adhesive materials, and in supramolecular adhesives, reversible bonds play a pivotal role. These bonds establish temporary yet strong interactions with the substrate, allowing the adhesive to conform to surface irregularities and form robust, adaptable attachments.

The effectiveness of interfacial adhesion depends on the type and density of reversible bonding sites within the adhesive. For example, hydrogen bonds can form rapidly with polar substrates, while π–π stacking interactions are particularly effective on aromatic or graphitic surfaces. Metal–ligand coordination, on the other hand, provides enhanced adhesion to substrates containing specific metal ions or ligands.^56^ Chemical motifs can assemble at the contact interface and further induce the aggregation status of the polymer network, providing exceptional adhesion properties.^57,58^ For example, the 2-uredio-4-pyrimidone (UPy) unit can form multivalent hydrogen bonds with the substrates, and in the meantime, the stacking of the UPy units, which has a planar architecture,^59^ can further reinforce the interfacial adhesion.^60^ Tethering UPy motifs to polymers or oligomers with a controlled molar ratio has been demonstrated as an effective way to make exceptional adhesion interfaces.^61^

One of the unique advantages of reversible bonds is their ability to dynamically respond to environmental stimuli,^30^ such as pH, temperature, or ionic strength. For instance, adhesives with pH-sensitive ionic bonds can selectively adhere or release based on the local acidity, making them ideal for biomedical applications like wound dressings or drug-delivery systems.^62^ Similarly, temperature-sensitive bonds provide reversible adhesion, where the adhesives can be easily removed or repositioned by heating.^63^ These dynamic interactions not only improve adhesion performance but also allow for reversible and reusable adhesives.^64^ Moreover, the combination of different bonding motifs can further expand the binding capability of the developed adhesives and motifs, allowing for more versatile application scenarios.^65^

### Effect of Polymer Assemblies on Adhesive Functionality

The structural organization of polymers within an adhesive system plays a decisive role in determining its mechanical strength, adaptability, and overall performance.^66^ Beyond the chemistry of individual bonds, the spatial organization, density, and alignment of polymer chains contribute significantly to the adhesives’ mechanical strength, flexibility, and responsiveness.

One of the primary ways that polymer assemblies affect adhesive functionality is through molecular interactions that govern adhesion and cohesion. In supramolecular adhesives, noncovalent interactions—such as hydrogen bonding, van der Waals forces, and host–guest interactions—facilitate dynamic bonding with surfaces while maintaining internal structural integrity.^67^ The spatial arrangement of polymer chains within the adhesive matrix determines the density and accessibility of these interactions, directly impacting adhesion strength and durability.^68^ Multiple mechanical gradients, such as stiffness or adhesion strength, can be incorporated into the adhesive design to improve the overall performance of adhesives.^69^ These gradients enable specialized functions, such as differential adhesion to multiple substrates or localized responsiveness to stimuli. Furthermore, polymer assembly can be dynamically controlled to include reconfigurable structures and customizable functions. For example, photocontrolled metallopolymer adhesives exhibit reversible adhesive properties upon cyclic light and heat treatments due to the precise control of the polymer status between assembly and disassembly.^70^

Additionally, polymer network architecture, whether linear, branched, or cross-linked, affects the viscoelastic properties and adaptability to different substrates. Highly cross-linked networks, either covalently or noncovalently, provide strong cohesion and mechanical robustness.^71^ The phase status of polymer assemblies also plays a pivotal role in adhesive performance. For example, in systems where phase separation occurs between hydrophilic and hydrophobic regions, surface adhesion can be tuned by manipulating polymer compatibility with the target substrate,^72^ which is particularly useful for bioadhesion and transdermal delivery.^73^

### Engagement of the Liquid Phase

However, the current research primarily focuses on the molecular design of the polymer networks or the chemistry of the dynamic bonds that govern the adhesive’s performance. While these aspects are undeniably important, they often overlook a critical element: the role of the liquid component within the adhesives.

In most designs, the liquid phase, typically water or another solvent, is treated as a passive medium, a mere carrier for the polymer network. This perspective simplifies an adhesive into a binary system of polymer and solvent, neglecting the intricate and potentially transformative interactions that occur between the polymer network and the liquid phase. These interactions, which include hydrogen bonding, hydrophobic effects, and electrostatic forces, are rich and highly tunable, offering a largely untapped avenue for extending the functionality of supramolecular adhesives.

For instance, the liquid phase can significantly influence the adhesives’ viscosity, diffusion properties, and responsiveness to environmental stimuli. Moreover, by engineering the chemistry and composition of the liquid phase, it is possible to modulate the dynamic behavior of the polymer network, enhance adhesion on diverse surfaces, or introduce additional functionalities such as antimicrobial activity or electrical conductivity.^74^

Recognizing and leveraging the interplay between the polymer network and the liquid component could open new dimensions in the design of supramolecular adhesives, transforming them from static systems into highly adaptable, multifunctional platforms. This shift in perspective requires a more holistic approach to adhesive design, one that integrates the liquid phase as an active and dynamic contributor rather than a passive solvent.

### Mucus as an Emerging Model in Designing Supramolecular Adhesives

Supramolecular adhesives feature tunable properties, self-healing capabilities, and responsiveness to environmental stimuli qualities that align closely with the behavior observed in biological systems like mucus. Mucus offers a natural blueprint for designing supramolecular adhesives due to its unique combination of a dynamic network and a functional liquid phase. The core differences of mucus-inspired supramolecular adhesives with other adhesives are the emphasis on the abundant functions incorporated by different liquid phase and their synergistic interactions with polymer networks. For example, compared to mucus-inspired supramolecular adhesives, traditional supramolecular adhesives focus on the mutual interactions originating from polymer networks and incorporated small molecules, whereas their functions are not well elucidated; and hydrogel-based adhesives are only prepared from the polymer networks with water, making them lack of abundant functions from other liquid phase, while the interactions between water and polymer networks are limited. Furthermore, the traditional supramolecular adhesives and hydrogel-based adhesives have been broadly discussed in previous reports, but there are still many unexplored interactions and functions for the mucus-inspired supramolecular adhesives ascribed to the richness of liquids in nature.

### Liquid Regulation of the Dynamic Network

#### 1 Diluting the Polymer Network

Variations in liquid concentration can significantly alter the behavior of the network. When the liquid content is high, the network becomes more pliable and less viscous. This is because the increased solvation reduces the density of cross-links within the polymer network, allowing the material to flow more freely. In mucus, this state is essential for functions such as lubrication and the clearance of foreign particles, where a lower viscosity facilitates movement. In synthetic systems, increasing liquid content can be used to create adhesives that are more adaptable to irregular surfaces.^75^ For example, hydrogels with a high water content are often employed in wound dressings or soft tissue interfaces where conformability and gentle adhesion are needed. Oil-loaded hydrogels with a hybrid interface can further reduce the adhesion, which is beneficial for developing medical gauze with low blood adhesion.^76^

Conversely, a reduction in liquid content increases the density of cross-linking within the dynamic network, leading to a stiffer and more robust material.^77^ This state is advantageous in scenarios that require stronger adhesion or structural integrity. In natural mucus, dehydration can enhance its protective barrier function by making it less permeable to external agents. Translating this principle to synthetic adhesives, reducing the liquid content can yield materials with higher mechanical strength and durability, suitable for load-bearing applications or environments with high mechanical stress.^78^

#### 2 Carrying Functional Molecules

**Figure 4:**
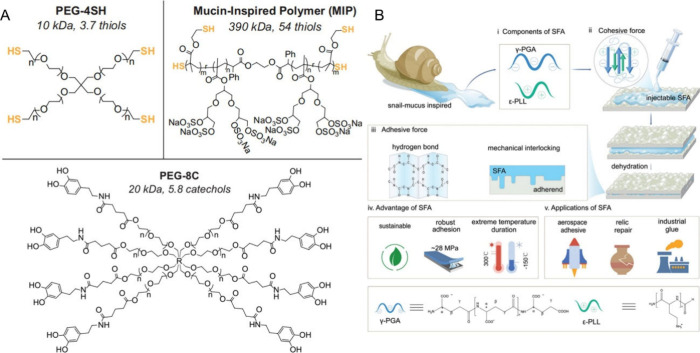
(A) Chemical structures of the thiol-functionalized polymers and catechol-functionalized polymers for the mucin-inspired adhesives are reprinted with the authors’ permission from ref (79). Copyright 2025, the Author(s). (B) Schematic diagram for the preparation, adhesion, advantages, and applications of the sustainable snail-inspired organic solvent-free adhesives. Reproduced with permission from ref (80). Copyright 2024 John Wiley and Sons.

Beyond acting as a bulk medium, the liquid phase in mucus contains functional molecules, such as ions, proteins, and small organic compounds, which actively regulate the behaviors of the dynamic network. These molecules interfere with or stabilize the dynamic bonds within the polymer network, enabling precise control over the material’s properties. For example, one biologically inspired adhesive using catechol–thiol bonding was recently developed, which integrated the multiple functions of mucus with strong and reversible adhesive properties in mussels (Figure 4A).^79^ The thiol-terminated and catechol-terminated PEG could be quickly entangled and reacted for gelation, showing tunable mechanical and adhesive properties at different ratios. The unique molecular mechanisms enabled the adhesives to have on-demand liquid-like or solid-like behaviors. Due to the intrinsic antifouling properties of mucin-inspired polymers, the adhesives demonstrated great application potential in tissue adhesion and antifouling coatings.

##### Ionic Regulation

Ionic interactions play a critical role in the cross-linking of mucin polymers. For example, divalent cations such as calcium (Ca^2+^) can bridge negatively charged residues on mucins, enhancing the network’s cohesion and mechanical strength. On the other hand, the presence of monovalent cations like sodium (Na^+^) or anions like bicarbonate (HCO^3–^) can compete with these interactions, weakening the cross-links and increasing the material’s flexibility.^81^ Synthetic adhesives inspired by this principle often incorporate ionic cross-linkers to achieve tunable mechanical properties.^82^ By adjustment of the ionic composition of the liquid phase, the adhesives can be made more rigid or more pliable, depending on the application.^83^

##### Small-Molecule Modulators

Small organic molecules in the liquid phase can also influence the dynamic bonding within the network. For instance, molecules with hydrogen-bonding capabilities can either stabilize the network by forming additional bonds or disrupt existing bonds, depending on their concentration and chemical affinity.^84^ This mechanism allows for reversible changes in the material’s properties, contributing to its adaptability. In synthetic systems, incorporating such modulators can enable the development of “smart” adhesives that respond to environmental stimuli. For example, adhesives that release small molecules in response to changes in pH or temperature could dynamically adjust their adhesion strength, making them suitable for applications like drug delivery or wearable sensors.^85^

#### 3 Regulating Phase Separation

Phase separation within mucus, driven by variations in liquid composition and mucin concentration, can lead to the formation of microenvironments with distinct chemical and physical properties. These microdomains influence mucus adhesion to epithelial surfaces, impacting processes such as wound healing, microbial colonization, and drug delivery. Understanding how the liquid phase contributes to these dynamic properties is essential for developing biomimetic materials, including supramolecular adhesives designed for biomedical applications. Typical examples like the sustainable snail-inspired solvent-free adhesives, which employed the liquid–liquid phase separation of two polypeptides [cationic ε-poly(l-lysine) (ε-PLL) and anionic poly(glutamic acid) (γ-PGA)] through electrostatic interactions and molecular entanglements (Figure 4B).^80^ Benefiting from phase separation, the adhesives demonstrated ultrahigh adhesive properties even under extreme temperature environment (−150 to +300 °C), while the adhesive properties were reversible and reusable, showing great values in many applications.

By carefully tuning the composition and concentration of the liquid phase, it is possible to achieve a wide range of behaviors from highly elastic and stretchable materials to rigid and load-bearing systems. This versatility is a direct result of the liquid’s ability to regulate the dynamic bonding within the networks, making it a key factor in the design of mucus-inspired materials. For instance, researchers have developed hydrogels that incorporate both hydrophilic and hydrophobic functionalities, allowing them to maintain strong adhesion in wet environments while being resistant to dissolution.^86^ Additionally, organogels use oil inclusion to resist water invasion and thus can achieve high adhesion in both dry and wet conditions.^87^ Such innovations highly rely on the liquid phase which maintains the material’s structural integrity and functionality.

### Synergy between the Dynamic Network and Functional Liquid

The regulation of the dynamic network by liquid components is a fascinating aspect of mucus’s functionality and adaptability. As discussed earlier, the liquid component is not merely a passive solvent but an active participant in the adhesive’s behavior. Hydration levels, ionic strength, and the presence of additives in the liquid phase can modify the polymer assembly and, consequently, the adhesive’s viscoelastic properties, adhesion strength, and environmental stability. Liquid in this context serves dual roles: as a bulk medium that contributes to the material’s physical properties and as a carrier for functional molecules that actively participate in tuning the network’s chemical and mechanical characteristics (Table 1). Understanding how liquid regulates the dynamic network sheds light not only on the adaptability of natural materials like mucus but also on how these principles can be applied in the design of synthetic adhesives.

**Table 1:** Overview of the Common Liquid Phase in Supramolecular Adhesives and Their Functionalities

	functional liquids in polymer networks	supramolecular interactions	potential functions in supramolecular adhesives
inorganic liquid phase	liquid metals (e.g., Ga, EGaIn, and Galinstan)	hydrogel bonding, metal–ligand coordination, dipole–dipole interactions	provide tunable mechanical and interfacial adhesive properties to the polymer networks; provide high electrical conductivity, high thermal conductivity, good fluidity, low evaporation rate, and excellent chemical stability to the polymer networks
	water	hydrogel bonding	hydrate the polymer networks for soft gels; provide mediums for cargo loading
organic liquid phase	ionic liquids (e.g., cationic midazolium, piridinium, ammonium, and phosphorium, with different counterions such as halides, tetrafluoroborate, and hexafluorophosphate)	hydrogen bonding, hydrophilic–hydrophobic interactions, electrostatic interactions, dipole–dipole interactions	provide high viscosity to obtain good mechanical properties; polymerizable with other molecules for customized molecular structures; provide extensive ion pairing for widely tunable mechanical, adhesive, flame-retardant, electrically and thermally conductive properties
	polyelectrolytes (e.g., PEDOT:PSS)	hydrogen bonding, electrostatic interactions, π–π stacking	provide tunable mechanical, electrical, and optical properties to the polymer networksprovide good processability, dispersibility, and stability to prepare functional polymer networks
	oils (e.g., essential oil, olive oil, silicone oil, and mineral oil)	hydrogen bonding, hydrophobic interactions	provide tunable mechanical and interfacial adhesive properties to the polymer networks; provide antimicrobial and hydrophobic properties to the polymer networks

The dynamic network of mucus, formed by mucin polymers, interacts intricately with the functional liquid phase to create a cohesive and versatile system. Key to this interaction are noncovalent forces such as hydrogen bonding, ionic interactions, and hydrophobic effects. These forces allow the mucin network to form reversible cross-links with the liquid phase, creating a structure that is both stable and adaptable. For example, hydrogen bonds between mucin chains and water molecules contribute to mucus’s viscoelastic properties, enabling it to deform under stress and recover its original structure without stress. Similarly, ionic interactions involving charged residues on mucins and dissolved ions in the liquid phase enhance the adhesive strength and stability of mucus.

In synthetic systems, recreating these interactions involves designing polymers with functional groups that can mimic the behaviors of mucins. For instance, incorporating hydrophilic groups such as hydroxyl or carboxyl groups into polymer chains can enhance hydrogen bonding with water molecules, replicating the dynamic behavior of mucin networks. Similarly, introducing ionic groups into the polymer structure can facilitate interactions with dissolved ions, mimicking mucus’s ability to adapt to environmental changes. Each type of interaction plays a distinct role in the synergy between dynamic networks and functional liquids. Hydrogen bonding provides the flexibility and reversibility that are essential for self-healing and adaptability. This is particularly important in applications where the adhesives must endure repetitive stress or environmental fluctuations. Ionic interactions contribute to the strength and durability of the adhesive by forming stable cross-links between polymer chains. Hydrophobic effects, on the other hand, help to create water-resistant regions within the adhesives, enabling them to function effectively in aqueous or humid environments.

The synergy between the dynamic network and functional liquid components is at the heart of mucus’s remarkable adhesive properties. This interplay allows for a combination of flexibility, durability, and adaptability, enabling mucus to perform critical biological functions across diverse conditions. Translating this synergy into synthetic supramolecular adhesives offers unique opportunities to develop materials that mimic the multifunctionality of natural systems. By dissecting the mechanisms that underpin this interaction, researchers can unlock new strategies for designing adhesives that replicate mucus’s ability to adhere to a variety of surfaces, even in challenging environments such as underwater or within the human body. Additionally, the self-healing and responsive properties of mucus provide a valuable model for developing adhesives that can adapt to mechanical stress or environmental changes.

### Emerging Applications of Mucus-Inspired Supramolecular Adhesives

Adhesives that can bind to diverse synthetic and biological surfaces in various environments have great technical implications. By leveraging the synergy between dynamic networks and functional liquids, mucus-inspired supramolecular adhesives hold the potential to revolutionize existing technologies while introducing novel solutions. With the consistent efforts of researchers from different fields, a range of mucus-inspired adhesive materials have been demonstrated. Their representative applications are summarized as follows.

### Essential-Oil-Based Supramolecular Organogel Adhesives

One of the most promising areas for mucus-inspired adhesives is in biomedicine. The ability of these adhesives to function effectively in wet environments makes them highly suitable for applications such as wound healing, tissue engineering, and drug delivery. Taking advantage of supramolecular chemistry to regulate molecular configuration and mechanical integrity,^88,89^ a variety of hydrogel-based supramolecular adhesives have been successfully developed, demonstrating individual or combined properties such as rapid and strong adhesion, reversible bonding, as well as enhanced mechanical strength, high toughness, and autonomous damage-healing abilities of the adhesives themselves.^90,91^

Compared to hydrogels, organogels offer broad-spectrum solvent selection and superior encapsulation ability for water-insoluble drugs.^92^ There are abundant synthetic or nature-derived bioactive oils that have been widely used in academia and industry.^93^ For example, plant-derived essential oils (EOs), recognized as “nature’s medicine” due to their superior biocompatibility, cost efficiency, and potent antibacterial properties.^94^ They demonstrated broad-spectrum bactericidal activities via multiple antibacterial mechanisms, thereby hindering the development of bacterial resistance.^95^ However, the limited solubility of EOs in aqueous solutions significantly restricts their practical application.^96,97^ Taking ideas from the design of mucus, involving those EOs in the preparation of organogel-based supramolecular adhesive has been demonstrated as an efficient way to overcome the above limitations and extend the application of supramolecular organogels.^98^

**Figure 5:**
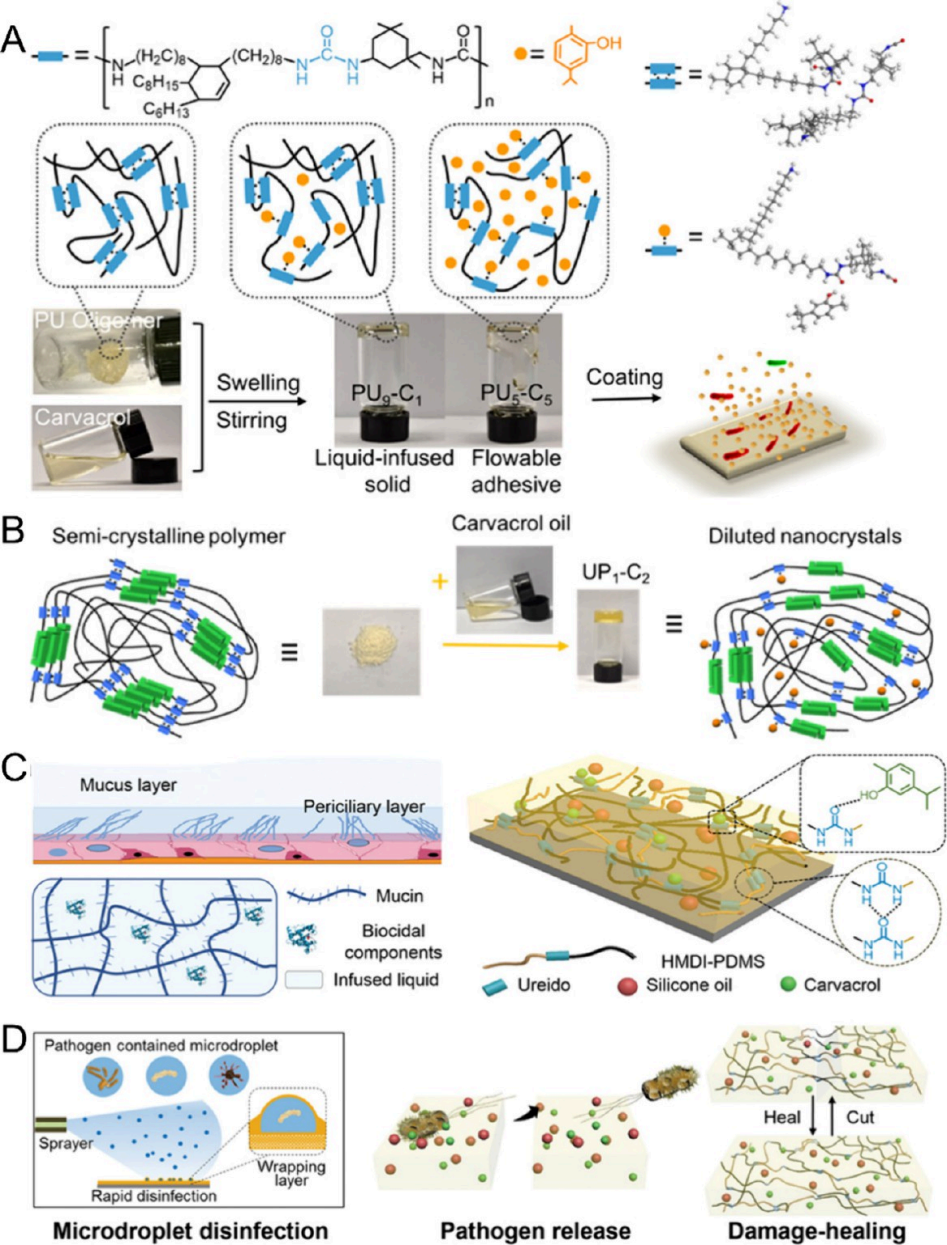
(A) Polyurea oligomers and (B) semicrystalline polymer were used to assemble with carvacrol oils for preparing different supramolecular antimicrobial organogel adhesives. Reproduced with permission from refs (74) and (87). Copyright 2020 and 2023 American Chemical Society. (C) Supramolecular slippery organogels demonstrated persistent fouling-release and damage-healing properties. (D) Antibacterial properties were enhanced by localized molecular controlled release with the wrapping layer. Reproduced with permission from ref (84). Copyright 2021 John Wiley and Sons.

Specifically, the polyurea oligomers and carvacrol oils (a type of EO) were coassembled through hydrogen bonding by swelling/stirring methods (Figure 5A).^74^ The obtained supramolecular adhesives presented a distinctive set of mucus-mimicking features, including oil-regulated mechanical properties, room-temperature processability, reusable adhesivity, and persistent bactericidal ability. Another kind of organogel-based supramolecular adhesives exhibited robust mechanical properties, controlled oil inclusion, and strong interfacial adhesion strength (Figure 5B).^87^ The encapsulated carvacrol oils could modulate the semicrystalline stackings into nanosized crystalline domains through intermolecular hydrogen bonding. These abundant hierarchical hydrogen bonds ensured strong interfacial binding, excellent mechanical properties, facilitated by the formation of nanosized crystalline domains. Besides, the controlled oil inclusion (50–500 wt %), glass transition temperature (2–80 °C), and storage modulus (0.12–93.56 MPa) ensure ease of processing and versatility for various applications in different antimicrobial and antiviral scenarios. The synergistic disinfection of microdroplets was achieved through the combined effect of lubricant liquid cloaking and the bactericidal activity of carvacrol oils, thereby enabling the spatial and local release of bactericidal molecules (Figure 5C,D).^84^ Moreover, the persistent fouling-release and self-healing properties will considerably increase the coating’s life-span.

With the rational selection of EOs and the polymer network, supramolecular adhesives with a combination of properties, such as antimicrobial, antiviral, dry and wet adhesion, as well as broad tunabilities on mechanical strength, adhesion strength and releasing profile can be developed. In those EO-based systems, the EOs serve not only functional components to bring new functions but also solvents to regulate the polymer network. Such design strategies would advance the fundamental understanding of the molecular regulation mechanism on the properties and performances of supramolecular materials.

### Flexible and Adhesive Electronics for Human–Machine Interfaces

**Figure 6:**
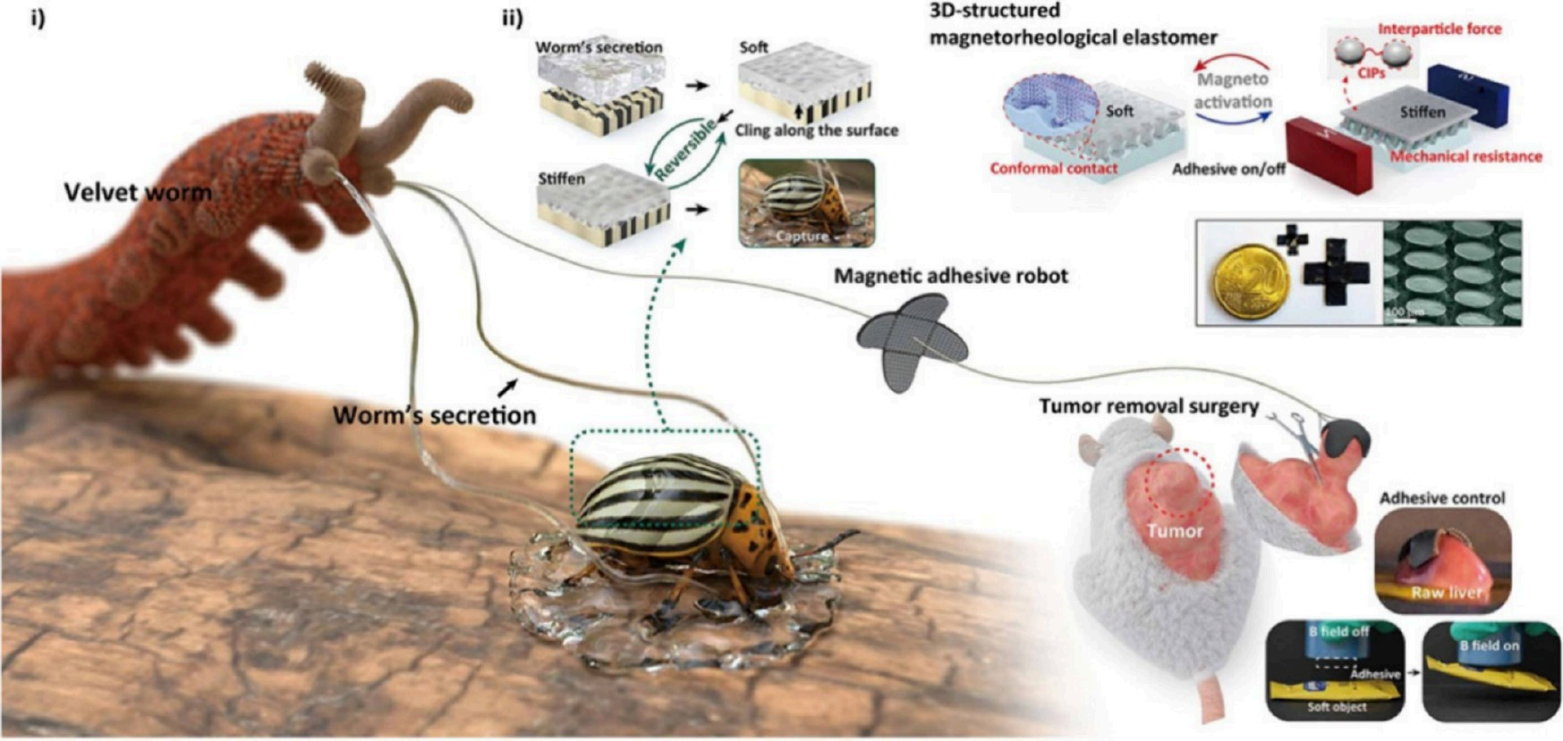
Schematics show that a velvet worm excretes mucus to capture prey, in which the mucus will be transformed from the soft state into a stiffened state when it contacts the prey. Inspired by the secretions, the adhesive robot was designed with a reversible magnetorheological effect to operate a tumor removal surgery. Adapted with permission under a Creative Commons License from ref (101). Copyright 2024 American Association for the Advancement of Science.

In the field of flexible electronics, the adaptability and self-healing properties of mucus-inspired adhesives make them suitable for bonding delicate components. These adhesives can maintain their functionality even under mechanical stress, ensuring the reliability of devices like wearable sensors and stretchable displays. Similarly, in robotics, the ability to create strong yet reversible bonds enables the development of robotic grippers and actuators that can interact with a variety of surfaces and materials. In particular, some bioinspired adhesives with stiffness-tunable interfaces have been recently achieved for controlled adhesive properties, showing precise adhesion and on-demand attachment/detachment to different substrates including wrinkled and soft biological and nonbiological surfaces.^99−101^ These advances may generate enormous influence on industrial development and medical operations. Based on the typical magnetorheological effect, these adhesives showed a quick response to transforming the modulus of their adhesive interfaces, endowing the traditional adhesives with intelligent behaviors. A typical example is the velvet worm-inspired adhesive robot; similar to the modulated modulus of the secretions when the velvet worm capture preys, the adhesive robot could control the elastic modulus of contact interfaces by remote magnetic control (Figure 6).^101^ Furthermore, the adhesive robot displayed stable transportation and negligible damage to soft and wet organs, showing great potential in future surgical operations.

**Figure 7:**
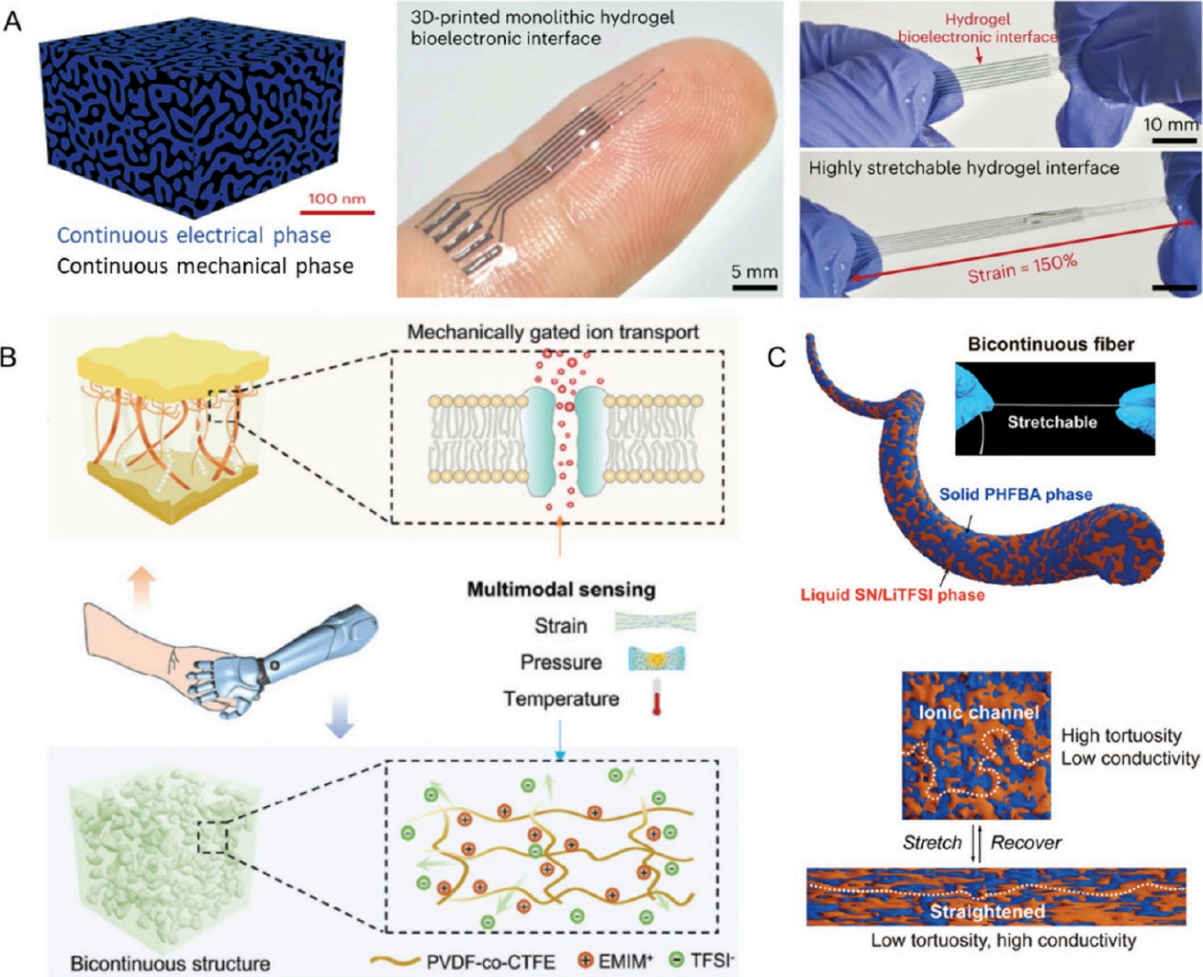
(A) The constructed conducting polymer hydrogel consisted of a continuous electrical phase and a continuous mechanical phase for applications in the self-adhesive bioelectronic interface. Reproduced with permission from ref (103). Copyright 2023 Springer Nature. (B) A schematic illustration of the microphase-separated bicontinuous ionogel with the capability of multimodal sensation is provided. Reproduced with permission from ref (105). Copyright 2023 John Wiley and Sons. (C) A schematic structure of the ionogel fiber with polymerization-induced bicontinuous phase separation is provided. Reproduced with permission from ref (106). Copyright 2024 John Wiley and Sons.

Supramolecular ionogels and ionic adhesives represent a rapidly developing area of materials science, driven by the emerging applications in human-machine interfaces.^102^ For instance, bicontinuous bioelectronic interfaces with continuous electrical phase and continuous mechanical phase were recently developed, which adequately employed phase-separated features to construct two different pathways in hydrogels (Figure 7A).^103^ Furthermore, the phase separation can enhance the adhesive’s ability to conform to rough or uneven surfaces, resulting in improved contact and stronger adhesion. At the same time, the liquid component contributes to the gel’s viscoelastic properties, allowing for a balance between flexibility and toughness. With well-defined molecule designs, the highly conductive adhesives simultaneously have other excellent properties, such as low modulus, high stretchability, and high tissue adhesion, which are all desired in flexible electronics, especially for wearable and implanted devices.^104^ Although promising, there are intrinsic limitations for the applications of hydrogel adhesives that their performance varies in response to the loss of water content.

Instead of water-based hydrogel or hydrogel adhesive, incorporation of ionic liquids into supramolecular polymer networks will result in the so-called ionogel or ionic adhesive.^107,108^ Unlike traditional hydrogel adhesives, ionic adhesives utilize ionic liquids as a functional liquid phase. This integration not only enhances the mechanical and adhesive properties of materials but also introduces additional functionalities that broaden the scope of potential applications. More importantly, the surface charge and chemical affinity of the ionic liquid will significantly affect the aggregation status of the host polymer network. Therefore, one of the key features of ionogels is the phase separation that occurs between the ionic liquid and the polymer network.^109^ This unique microstructure can significantly influence the material’s interfacial adhesion and mechanical strength. For example, incorporating the midazolium-based ionic liquid into the poly(vinylidene fluoride-*co*-chlorotrifluoroethylene) (PVDF-*co*-CTFE) can obtain a microphased-separated ionogels, for which the ionic liquid-rich phase contributes to the ionic conductivity and the polymer-rich contributes to the elasticity (Figure 7B).^105^ Such ionogel shows excellent elasticity and multimodal sensation through the ion-driven stimuli–electricity conversion, which is caused by the directionally flow of the ionic liquid within the polymer network under various mechanical or temperature changes. Similarly, the application of bicontinuous design into fibrous materials would also harvest the unique features of ionogels, making the ionogel fiber excellent candidates for the iontronic applications (Figure 7C).^106^

In addition to high ionic conductivity, the inclusion of ionic liquids also imparts, in some cases, ion selectivity,^110^ which are properties rarely achievable with conventional adhesives.^111^ These features open up new possibilities for applications in human–machine interfaces, such as wearable electronics, soft robotics, and bioelectronic devices.^112^ For example, the high ionic conductivity of these materials enables efficient transmission of electrical signals, making them ideal for flexible sensors and actuators. Additionally, the ion selectivity of certain ionogels can be leveraged for applications in ion transport membranes or electrochemical devices.^113^

Despite these promising attributes, the development of supramolecular ionogels and ionic adhesives is still in its early stages. Current research is focused on understanding the complex interplay between the polymer network, ionic liquid, and external stimuli, as well as optimizing their design for specific applications. With further exploration, these materials have the potential to redefine adhesive technologies and expand their functionality into emerging fields.

### Therapeutic Supramolecular Adhesives

Supramolecular adhesives are emerging as a cutting-edge platform for tissue repair and drug delivery, offering dynamic and multifunctional solutions for biomedical challenges. These materials are particularly well-suited for creating patches or adhesives capable of adhering to wet and soft tissues while simultaneously loading and releasing therapeutic agents or living cells in a controlled manner.

**Figure 8:**
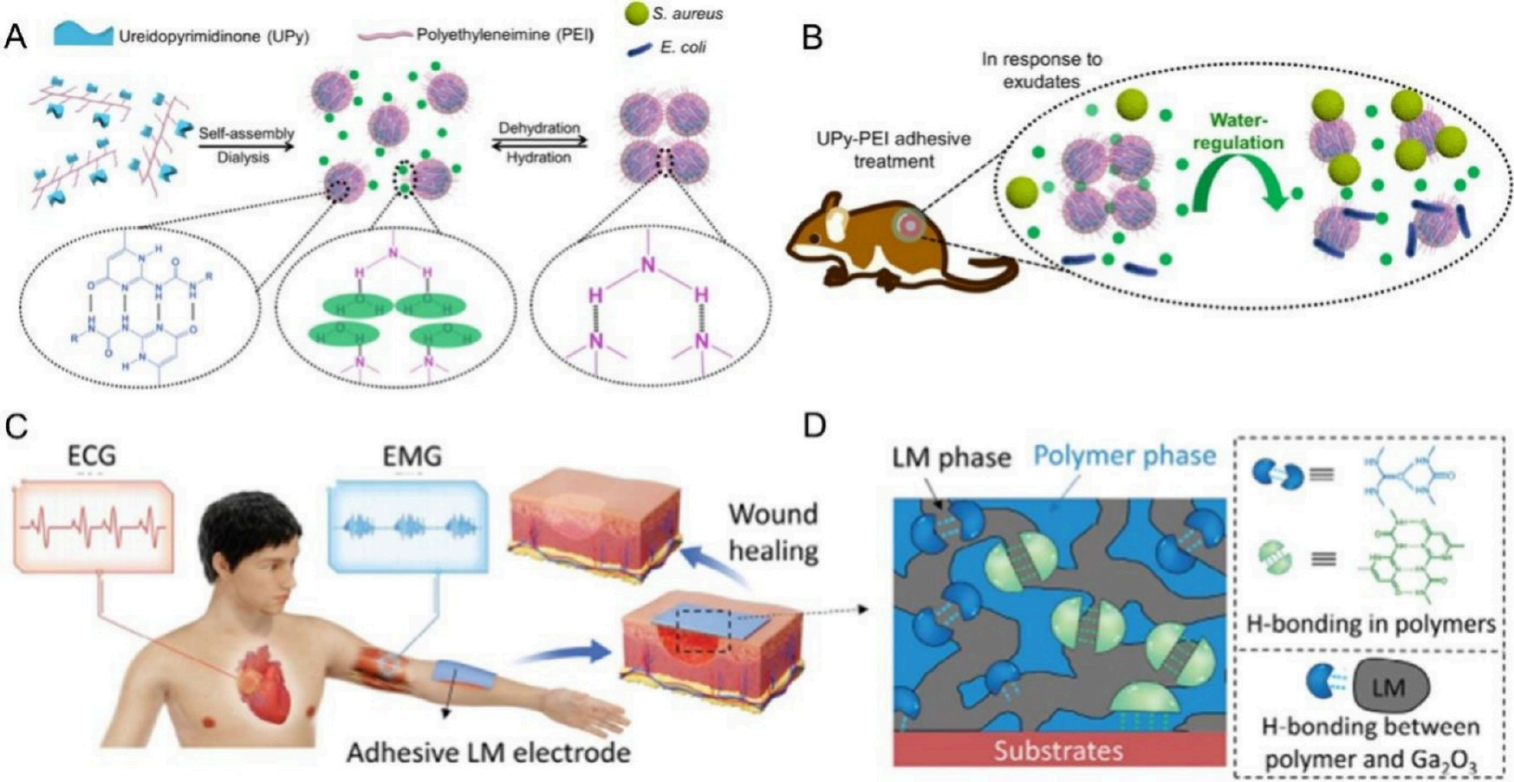
(A) A supramolecular adhesive was assembled and aggregated by hydrogen-bonded UPy–PEI particulates, showing reversible hydration and dehydration behaviors. (B) The particulate-aggregated adhesive exhibited a sustained release ability for wound disinfection due to the superior adhesive and exudate-sensitive properties on mice wound sites. Reproduced with permission from ref (114). Copyright 2020 American Chemical Society. (C) The LM-enhanced adhesive patch achieved tunable adhesive properties for monitoring electrophysiological signals and promoting wound treatment. (D) The material structure of LM-enhanced UPy–PEI adhesive patch is provided. Reproduced with permission from ref (115). Copyright 2024 John Wiley and Sons.

A key advantage of supramolecular adhesives lies in their dynamic nature, which enables strong adhesion to wet, irregular, or moving surfaces like internal organs, without causing damage to the tissue. The adaptive interface ensures that the adhesive can conform to the tissue surface, maintain stable attachment, and adapt to physiological movements. Moreover, taking advantage of the abundant supramolecular interactions, a variety of adhesives and patches with controlled properties for different therapeutic applications have been prepared.^114−118^ According to the demands in specific application scenarios, molecules can be delicately tailored to meet the functional requirements. In particular, multivalent UPy motifs have been widely adopted to be grafted onto various molecules for diverse supramolecular adhesives and patches. For example, UPy motifs were grafted onto polyethylenimine (PEI) backbones to prepare a particulate-aggregated adhesive (Figure 8A).^114^ This fluid-like supramolecular UPy–PEI adhesive integrated strong quadruple hydrogen bonds from the UPy motifs with weak hydrogen bonds from the PEI chains, resulting in dynamic and reversible properties to regulate the mechanical, adhesive, and antimicrobial abilities. Apart from these excellent properties, the assembled and aggregated hydrogen-bonded particulates enabled the adhesive to have exudate-sensitive behaviors, ascribed to the dissociation of hydrogen bonding by water molecules, which facilitated the sustained release of its own antimicrobial particulates to treat the bacteria-infected wound sites (Figure 8B). All of the demonstrated properties suggest supramolecular patches ideal for localized therapies, where precise placement and retention are critical. Following the UPy–PEI adhesive, leveraging the advantages of liquid metal (LM) such as high mobility and conductivity,^119,120^ a LM-enhanced therapeutic patch was developed, realizing a combination of high and stable conductivity, tunbale and adaptive tissue adhesion, good tissue compatibility, degradability, and recyclability.^115^ The developed patch further incorporated the weak yet abundant interfacial interactions between the UPy–PEI molecules and LM to regulate all of the aforementioned properties, endowing the multifunctional adhesive patch with on-demand functions for different applications such as electrophysiology monitoring and wound treatment (Figure 8C). Based on these advancements, the hierarchical hydrogen bonds are deemed as important tools for performance development in an functional adhesive patch, as which can be customized by simply tuning the proportion of LM and UPy–PEI molecules (Figure 8D). All of these developments benefitted from the molecular or material engineering promise that the diversified supramolecular interactions can bring rich and demanded material properties for many therapeutic applications.

In addition to their adhesive properties, these materials can be engineered to act as drug or cell delivery vehicles. Therapeutic agents, including small molecules, peptides, or even delicate biologics like vaccines, can be encapsulated within the polymer network or interspersed in the adhesive matrix.^121^ The dynamic bonds within the adhesive allow for controlled release triggered by environmental stimuli such as pH, temperature, or enzymatic activity.^122−125^ For example, supramolecular adhesives have been developed to release vaccines over time, enhancing immune response while minimizing the need for repeated doses.^126^

Similarly, supramolecular adhesives are being explored for cell therapy applications. These materials can encapsulate living cells, such as stem cells or immune cells, within their networks, providing a protective yet bioactive environment.^127^ The controlled rheology of the supramolecular adhesives renders them readily for injection and localized adhesion.^128^ Once attached to the target tissue, the adhesives can facilitate localized cell delivery and promote tissue regeneration through direct cell activity or by releasing bioactive factors. The ability to precisely control the release of cells or drugs makes these adhesives a valuable tool for advanced therapeutic interventions.

With their unique combination of strong adhesion, dynamic reversibility, and multifunctional capabilities, supramolecular adhesives hold immense potential for revolutionizing tissue repair, vaccine delivery, and cell therapy. These systems represent a promising step toward more effective, targeted, and patient-specific therapeutic solutions. Despite these advancements, challenges persist in optimizing the balance between adhesive strength, biocompatibility, and release kinetics. Long-term stability, immune response, and scalability for clinical use remain areas of active research. Additionally, ensuring the viability of encapsulated cells and the stability of delicate therapeutics during fabrication and application requires further refinement.

### Challenges and Outlook

The development of mucus-inspired supramolecular adhesives is a rapidly advancing field with immense potentials. However, to fully realize the promise of these materials, several challenges must be addressed, and future research must continue to refine our understanding of their underlying mechanisms. By exploring innovative approaches and overcoming current limitations, researchers can unlock new possibilities for these adhesives in biomedicine, industry, and beyond.

### Challenges in Mimicking Biological Systems at a Molecular Level

While mucus serves as an excellent source of inspiration, replicating its properties in synthetic systems presents significant challenges. One major obstacle is achieving the same level of complexity and functionality found in natural mucus. The interplay between the dynamic network and the functional liquid in mucus is highly coordinated, and reproducing this synergy synthetically requires precise control over molecular interactions. Scalability and cost-effectiveness are also concerns, as the processes involved in synthesizing supramolecular adhesives can be resource-intensive. Despite these challenges, advances in material science and molecular engineering continue to bring researchers closer to creating synthetic adhesives that rival the performance of their biological counterparts.

### Advancing the Understanding of Mucus’s Molecular Mechanisms

A deeper understanding of the molecular interactions within mucus is essential for optimizing its synthetic counterparts. While significant progress has been made in characterizing the dynamic networks and functional liquid components of mucus, there remain gaps in our knowledge regarding how these elements interact under various environmental conditions. For instance, the precise role of ionic gradients and pH in modulating mucus’s adhesive properties is not yet fully understood. Addressing these gaps will require advanced analytical techniques, such as high-resolution imaging and molecular simulations, to study mucus at the nanoscale.

### Improving Scalability and Cost-Effectiveness

One of the primary challenges in translating mucus-inspired adhesives from the laboratory to real-world applications is scalability. The complex processes involved in synthesizing these adhesives, including the precise control of noncovalent interactions, can be resource-intensive and difficult to reproduce on an industrial scale. Additionally, the cost of the raw materials and the specialized equipment required for production often exceeds that of conventional adhesives. Future research should focus on developing streamlined synthesis methods, including the continuous flow synthesis, microwave-assisted synthesis, ultrasound-assisted synthesis, solid-phase synthesis, and solvent-free synthesis. Compared to existing polymerization methods such as solution polymerization and emulsion polymerization, these emerging synthesis methods may provide significant advantages on controlling the reaction parameters, simplifying the separation and purification, and improving the product yields and purities. Specifically, the continuous flow synthesis allows the reactants to be continuously pumped through a reactor in a flowing stream for precise control; the microwave/ultrasound-assisted synthesis utilizes external energy to accelerate the reaction and reduces the products with nonuniform properties; the solid-phase synthesis renders the production to be easy of purification and have a high reproducibility; and the environmentally friendly solvent-free synthesis makes the production process easy to scale up. It is also worthy to explore alternative, cost-effective materials that can replicate the behaviors of mucus. For example, biological polymers derived from renewable sources could offer a more sustainable and economical solution.

### Necessity for Cross-Disciplinary Collaboration

The challenges and opportunities in this field highlight the need for cross-disciplinary collaboration. Insights from biology, chemistry, physics, and engineering are all essential for advancing the design and application of mucus-inspired adhesives. For example, biologists can provide a deeper understanding of mucus’s natural functionality, while chemists can develop new molecular frameworks to replicate these properties. Engineers, in turn, can optimize the production processes and integrate these adhesives into practical devices. Such collaborations will be key to overcoming current limitations and unlocking the full potential of these materials.

Ultimately, mucus-inspired supramolecular adhesives exemplify how nature can guide technological advancement. By learning from biological systems that have evolved over millions of years, scientists can create materials that are not only efficient but also harmonious with the environment. As research continues to advance, these adhesives are poised to redefine what is possible in adhesive technologies, offering solutions that are as adaptable and dynamic as the natural world itself.
